# Adapting a school readiness intervention for children with sickle cell disease: an intervention mapping for adaptation approach

**DOI:** 10.3389/fpubh.2026.1862943

**Published:** 2026-07-06

**Authors:** Andrew M. Heitzer, Eunyoung Kang, Mollie Tamboli, Marshetta Brazley-Rodgers, Anne Kolb-Quinn, Yvonne Carroll, Heather M. Conklin, Allison A. King, Jane S. Hankins, Katherine Pears, Maria E. Fernandez

**Affiliations:** 1 Psychology and Biobehavioral Sciences, St. Jude Children’s Research Hospital, Memphis, TN, United States; 2 Institute for Implementation Science, University Texas Health Science Center at Houston, Houston, TX, United States; 3 School Program, St. Jude Children’s Research Hospital, Memphis, TN, United States; 4 Department of Hematology, St. Jude Children’s Research Hospital, Memphis, TN, United States; 5 Departments of Occupational Therapy, Neurology, and Pediatrics, Washington University in St. Louis, St. Louis, MO, United States; 6 Global Pediatric Medicine, St. Jude Children’s Research Hospital, Memphis, TN, United States; 7 Oregon Social Learning Center, Eugene, OR, United States

**Keywords:** adaptation, caregivers, children, developmental, intervention mapping, preschool, school readiness, sickle cell

## Abstract

**Background:**

Preschool children with sickle cell disease (SCD) are at high risk for early and progressive neurocognitive deficits that negatively affect academic outcomes. Preschoolers with SCD are more likely to display deficits in school readiness skills such as early literacy, numeracy, and self-regulation. Previous behavioral interventions for children with SCD have displayed highly variable feasibility and adherence. The goal of this study was to formally adapt an evidence-based school readiness intervention for children with SCD.

**Methods:**

The Intervention Mapping for Adaptation (IM Adapt) methodology was used to adapt an intervention for patients with SCD and their families. IM Adapt is a systematic and iterative problem-solving approach to adapt health promotion interventions for specific populations. The evidence-based school readiness intervention Kids in Transition to School (KITS) was identified through the IM Adapt strategy. This intervention showed preliminary fit with the needs of children with SCD. A planning group composed of providers, subject matter experts, and caregivers of patients with SCD was established to complete the adaptation of KITS for this population.

**Results:**

We added content to KITS describing how to respond to a child’s pain episode, the impact of sleep disruptions on school performance, medical accommodations offered to children with SCD in the school setting, and how to establish school services. SCD-related content included discussion questions to facilitate group problem-solving and peer support. We converted the core child-directed content into animated videos for the children and their caregivers to watch at home together. We also provided a school readiness workbook to all families to cover early literacy and numeracy topics. We updated the parent- and child-directed intervention materials to improve cultural representation and include books that promote cultural pride for African American populations.

**Conclusion:**

By working with families and community stakeholders, we adapted an evidence-based interventions that may address barriers and health inequities. IM Adapt provides a formal process in which to document and evaluate these adaptations.

**Clinical trial registration:**

https://www.ClinicalTrials.gov, identifier NCT06367192, 4-11-2024.

## Background

Sickle cell disease (SCD) is a genetic disorder that affects hemoglobin and is characterized by early and progressive neurocognitive dysfunction. In the United States (US), SCD predominantly affects individuals of African descent (1, 2). Slowed development is thought to result from a combination of social determinants (3) and disease-related biologic factors (4, 5). Patients with SCD experience greater rates of poverty and fewer protective socioeconomic factors when compared with the Black population in the US (6), and these factors contribute to long-term negative neurocognitive outcomes (5, 7). Meta-analytic findings indicate that even in the absence of a silent cerebral infarct (SCI) or overt stroke, patients with SCD perform approximately two-thirds of a standard deviation below controls (healthy or sibling) across neurocognitive domains (8).

At St. Jude Children’s Research Hospital (St. Jude), 73% of preschoolers with SCD lack basic school readiness skills (9), including early literacy, numeracy, and self-regulation skills. By adolescence, 27% of patients with SCD at St. Jude display intellectual impairment (<5th percentile) and 30% of patients were retained in grade (7). Neurocognitive deficits contribute to difficulties transferring to and remaining in adult healthcare (10, 11), along with high rates of unemployment that approach 50% in patients with SCD in the US (12, 13). Ultimately, patients with SCD and neurocognitive deficits have a worse health-related quality of life (14–16).

Preschoolers with SCD show notable weaknesses in pre-academic skills (17–19) and aspects of executive functioning, particularly self-regulation (20, 21). These fundamental skills are the strongest predictors of academic success in elementary (22, 23) and high school (24) in the general population. School readiness skills and self-regulation are highly malleable (25–27), and early intervention produces the greatest gains (28, 29).

Recognizing the profound impact of neurocognitive dysfunction, recent American Society of Hematology cerebrovascular guidelines endorsed the implementation of neurocognitive rehabilitation strategies in patients with SCD (30). However, to date, there have been limited attempts to address these patients’ neurocognitive and academic deficits. Previous intervention studies have included disease modifying or curative treatments (7, 31, 32), neuropharmacological approaches (33, 34), computerized interventions involving repetitive drill practice (35, 36), and behavioral approaches teaching parents and/or patients new skills (37, 38).

SCD treatments such as hydroxyurea may slow neurocognitive decline, but neurocognitive deficits persist and appear to worsen with age (7). Neurocognitive outcome data after curative stem cell transplantation or gene therapy are limited but suggest that neurocognitive performance may stabilize or improve (39). Curative approaches are unavailable for most patients with SCD due to prohibitive costs and treatment-associated risks (40). Pilot studies of methylphenidate (33) and memantine (34) suggest potential benefit, but were limited by small samples and metrics of feasibility, acceptability, and adherence were not reported.

Computerized or behavioral approaches have shown some potential to improve neurocognitive or academic outcomes in patients with SCD, but these approaches have poor feasibility. A home-based computerized working memory intervention, Cogmed (35), had low adherence, with only 15% of participants completing all recommended sessions. In an adolescent/adult executive function intervention, fewer than half of those approached consented, and only half of those completed the required sessions—though completers did show improvements (37). These findings suggest that behavioral and computerized interventions can improve outcomes, but feasibility barriers must be addressed.

Among the SCD population, an intervention targeting the parent–child relationship, knowledge of development, and language acquisition (Parents as Teachers), has been piloted in two studies (41, 42). Across these studies, the program demonstrated strong feasibility (>50% approached families consented) (43) and was associated with improvements in neurocognitive and language development (38). However, attendance was highly variable across both studies. This intervention was not adapted for families of children with SCD and did not include any content specific to this patient group. In the general population, evidence-based interventions to improve self-regulation and school readiness in preschoolers exist (6, 44). These school readiness interventions incorporate many of the same principles as the Parents as Teachers intervention but emphasize the skills needed to successfully transition into a formal school setting (e.g., self-regulation, early numeracy and literacy) for preschoolers (ages 3 + years). No studies have evaluated the adaption of a school readiness intervention for children with SCD.

Addressing barriers to adherence and increasing accessibility is critical to intervention success (45). This is particularly true for patients with SCD, who are predominantly racial minorities from underserved communities (46). These families are less likely to report developmental concerns (47), experience greater stigma for seeking help (48–50), have less confidence that interventions will be beneficial (51), and underuse early intervention services for children with developmental delays (45). Families of children with SCD experience both structural and interpersonal racism that interfere with service provision (52, 53). Although these barriers limit the use of many interventions, they represent potential intermediate targets for families with SCD. By working with families and community stakeholders, we can adapt an evidence-based intervention to address barriers and health inequities and strengthen the implementation of services (45). Therefore, using the Intervention Mapping for Adaptation (IM Adapt) methodology, we sought to adapt an evidence-based school readiness intervention, Kids in Transition to School (KITS) (54), for preschool children with SCD. This paper describes how IM Adapt can be used to systematically adapt an intervention for a target population, with the goal of highlighting the application of this approach and providing a template for future adaptation efforts.

## Methods

### Intervention mapping for adaptation

IM Adapt is a systematic and iterative tool used to adapt evidence-based interventions for specific populations and context (Table 1) (55). IM Adapt is an extension of Intervention Mapping, a planning approach that is based on using theory and evidence to develop interventions to address health problems and promote community participation (56, 57). Intervention Mapping and IM Adapt follow similar steps, but Intervention Mapping develops an intervention from the ground up. In contrast, IM Adapt starts with an existing intervention and uses a series of six steps to understand the logic of the evidence-based intervention, including the core components, and then adapts it based on a comparison with the needs of the new population and setting.

**Table 1:** Intervention mapping for adaptation tasks.

Tasks	Sub-tasks
1. Conduct needs assessment	Conduct a needs assessment and develop a logic model of the problem Develop a logic model of change Describe organizational capacities and goals Write program goals
2. Search for evidence-based interventions	Search for interventions to address the problem Judge basic fit
3. Assess fit and plan adaptations	Judge behavioral and environmental fit Judge determinants and change methods fit Judge delivery, design, and cultural fit Judge implementation fit Identify essential elements of the intervention
4. Make adaptations	Prepare design document for adaptation Pretest adapted materials Produce final adaptations
5. Plan for implementation	Identify potential implementers, implementation behaviors, and outcomes Develop implementation and maintenance scope, sequence, and instructions Plan activities to motivate and train for implementation
6. Plan for evaluation	Use logic model to write evaluation questions Choose indicators and measures Choose evaluation design Plan data collection, analysis, and reporting

### Health equity implementation framework

An important step for any adaptation effort is to clearly identify the needs of the priority population. A modified version of the Health Equity Implementation Framework (HEIF) was used to better understand the needs of families with preschool children with SCD (58). This determinant framework emphasizes barriers and facilitators at all levels of implementation, including the patient/caregiver, provider, clinical encounter, intervention, and healthcare system. The HEIF combines two conceptual frameworks: one framework focuses on implementation science (59), and the other focuses on healthcare disparities (60). Combining these frameworks addresses implementation problems in a way that integrates health equity concerns and accounts for influencing factors at multiple levels, particularly those unique to vulnerable patient populations (e.g., disparate clinical care).

### Setting

The primary setting of the adaptation was a large children’s hospital and academic research center located in Tennessee. The hospital serves children who have catastrophic diseases, including over 800 patients with SCD. The hospital provides comprehensive treatment for children with SCD, including comprehensive medical care, case management services, a full pharmacy, patient advocacy, and psychosocial support for patients and their families. The hospital has a school program and school coordinators. These coordinators serve as community school liaisons and advocate with families for the educational needs of patients who need extra support.

### Planning group

To facilitate the IM Adapt process, a planning group was assembled. The group was composed of expert researchers in SCD (JSH and AAK), neurocognitive interventions (HC), and intervention adaptation (MEF and EK). Other members of the group included the developer of the original intervention (KP), a hematology administrator/community liaison (YC), a preschool teacher (AKQ), a school coordinator (MBR), a psychology research associate (MT), and two patient caregivers. Small and large group meetings were held throughout the adaptation phases. In addition to discussions among caregivers and stakeholders as a part of the planning group, preliminary findings and decisions were shared with community members through newsletters and presentations to local groups serving the SCD community to maintain engagement.

### Task 1: Perform needs assessment, assess organizational capacity, and create logic models

A logic model of the problem was developed by the planning group. The priority population’s needs and assets were evaluated through a literature review, previous experiences with the priority population, and our existing qualitative data on determinants that influence access to early intervention services for children with SCD based on caregiver (61) and provider perspectives (62). Together, this informed the preliminary logic model. The literature review was primarily used to describe the strengths and weaknesses of previous behavioral interventions and determinants that influence the participation of this patient population. During the organizational capacity assessment, a group of individuals who could implement the adapted intervention within the hospital’s school program were identified, and their roles as interventionists were established. These individuals expressed a strong interest in learning about the program and working with the SCD population and were trained in child development and education, consistent with the original KITS facilitators. Available spaces within the hospital where programming could take place were identified and approved by hospital leadership.

Based on the initial needs assessment and logic model, a broad search for school readiness interventions was conducted using published systematic reviews (63, 64). The KITS (54) intervention was chosen based on the initial fit, and the original needs assessment and logic model of the problem for KITS was obtained. The needs assessments and logic models for KITS were compared with those developed for our setting/population. This comparison revealed knowledge gaps that required further assessment. Specifically, these gaps involved health equity determinants (e.g., access to care, cultural factors), desired intervention content, intervention delivery/access, organizational capacity, and community participation.

Therefore, a more comprehensive needs assessment was conducted, including semi-structured interviews and surveys with families of young children with SCD and stakeholders within the hospital system and broader community. Fifteen interviews (65) were conducted with caregivers of young children with SCD. Interviews were audio-recorded, transcribed, coded, and analyzed. The initial guides, coding, and analyses were based on the HEIF and also addressed the knowledge gaps noted above (58). Further, caregivers completed questionnaires assessing their knowledge of child development and potential psychosocial barriers to care. The interview guides used to collect this data have been published elsewhere (65).

As previously reported (65), medical/educational providers and caregivers of children with SCD (ages 4–6) were recruited in Memphis, TN. Snowball sampling identified providers familiar with developmental needs of young children with SCD, beginning with St. Jude Children’s Research Hospital and a local early childhood provider. These providers referred colleagues with experience in developmental services and/or working with families of young children with SCD. Caregivers were recruited via the Sickle Cell Clinical Research and Intervention Program (SCCRIP), a longitudinal cohort of patients with SCD (66). Eligible caregivers were English-speaking and had a child (ages 4–6) with any SCD genotype enrolled in the cohort. Fifteen providers (5–29 years’ experience) across medical, education, and advocacy sectors completed interviews. Roles included hospital-school coordinators (*n* = 4), hospital teachers (*n* = 2), school leaders (hospital and community; *n* = 4), early childhood health/disability specialists (*n* = 2), and one adult SCD advocate, hematologist, and hematology administrator. Seventeen caregivers enrolled; 15 completed surveys/interviews (2 lost to follow-up). Caregivers were ages 23–40 (M = 29.4), primarily female (94%), and all identified as Black/African American.

After coding was completed, the primary themes and survey data for the caregivers and stakeholders were presented to the planning group. These data were repeatedly referenced across multiple planning group meetings to complete Steps 2–6.

### Task 2: Search for evidence-based interventions and judgement of basic fit

The search for an evidence-based school readiness intervention occurred before a thorough needs assessment was conducted (Task 1). This is common in adaptation efforts and is one of the pathways described in IM Adapt. A close collaboration with the developer of the original intervention was maintained to identify knowledge gaps throughout Task 1. The basic fit of the KITS program with the needs of our setting and organizational capacity was continuously evaluated.

### Task 3: Assess fit and plan adaptations

Intervention materials from the KITS program were organized into their individual intervention components for analysis of the extent to which the existing intervention fit the needs of the new population and setting. The change model from KITS was compared to the change model developed by the planning group to evaluate the fit between the outcomes and determinants from the original intervention. The appropriateness of the change methods (i.e., techniques for influencing changes in determinants of behaviors) was evaluated based on the identified determinants of behaviors and environmental outcomes (57, 67). Intervention manuals, implementation procedures, and scientific articles were reviewed. Based on caregiver and provider data, the delivery, design, cultural, and implementation fit of KITS with the SCD population in our setting was evaluated. The essential components of the intervention that needed to be maintained during the adaptation process were identified. For each fit category (Table 2), discrepancies between the change models and original intervention were discussed by the planning group and rated as having “good,” “adequate,” or “poor” fit. The primary source of data (e.g., caregiver or stakeholder interviews) informing these ratings is listed in Table 2. Broadly, caregiver and stakeholder data was considered and integrated concurrently based on the themes that were generated during qualitative analyses (65). These ratings were used to summarize the extent of adaptations needed to tailor each category to the new population and setting. Based on the mixed methods data and literature review presented to the planning group, adaptation ideas were generated in smaller group discussions guided by prompts from the IM Adapt framework and then later presented to the larger group for voting. All proposed adaptations required unanimous approval for inclusion in the initial set of adaptations (Table 2).

**Table 2:** Assessment of the intervention fit and initial adaptations.

Fit category	Fit	Primary data sources	Rationale	Adaptations
Health promoting behaviors for child	Adequate	Literature review and stakeholder interviews	Health promoting behaviors for the child are largely the same. However, KITS does not directly address the medical complications experienced by patients with SCD.	Incorporate content for children about how to communicate with their teacher or school staff about pain, fatigue, and discomfort.
Health promoting behaviors for caregiver	Adequate	Stakeholder interviews	KITS teaches parents to promote school readiness skills known to be weaker among children with SCD. Content is missing to address ways in which parents can communicate with schools about their child’s medical needs.	Incorporate content for parents about how to advocate for their child’s medical needs in a school setting and strategies to adhere to child’s medical treatment.
Change methods for children	Good	Literature review	Change determinants for KITS child sessions include knowledge, skills, and attitudes. These determinants are all highly consistent with the adapted logic model of change.	None
Change methods for caregivers	Good	Caregiver interviews	Change determinants for KITS parent sessions include attitudes, self-efficacy, knowledge, outcome expectations, and skills. These change determinants remained highly relevant based on data collected from caregivers and providers.	None
Component delivery—children	Poor	Caregiver and stakeholder interviews	Caregivers consistently reported that there were significant barriers to attending in-person classes with their children.	Core content from the child classroom sessions was made into videos for children to review with their parents at home.
Component delivery—caregivers	Adequate	Caregiver interviews	Caregivers preferred meeting virtually in groups so that they could learn from other caregivers and build a community of families with shared experiences.	Caregiver sessions were delivered virtually.
Cultural relevance	Poor	Review of intervention materials and stakeholder interviews	KITS intervention materials (handouts and books) were not representative of the demographic/cultural makeup of the SCD population at our institution.	The handouts for parents were updated to be more culturally representative, and the books given to children were changed to include more culturally representative characters and promote cultural pride.
Implementation plan	Adequate	Caregiver interviews	Caregivers expressed interest in learning from other caregivers with similar experiences in addition to trained professionals. The intervention setting (hospital vs. school) differed.	Caregivers of children with SCD are trained to co-facilitate the group parent sessions. Families are recruited from a pediatric hospital setting.

KITS, kids in transition to school; SCD, sickle cell disease.

The meaning of each “Fit” rating is dependent on the category, and the specific changes needed are highlighted in the Rationale and Adaptations columns. “Good” fit indicates that the original intervention component aligns well with the new target population and context, requiring no changes or only minor, surface-level adjustments (e.g., wording or formatting). “Adequate” fit indicates partial alignment; the intervention component requires moderate adaptation to improve relevance or feasibility (e.g., modifying delivery methods, adjusting content emphasis, or tailoring materials), while the core function remains intact. “Poor” fit indicates limited or no alignment; the intervention component requires substantial modification or replacement, involving changes to core elements such as content, delivery approach, or underlying assumptions to be appropriate for the new context.

### Task 4: Make adaptations

A list of planned changes was created, and the location of the change in the intervention materials was established. Initial drafts of the changes were developed and circulated among the planning group for feedback. The changes were then iteratively updated over several rounds of feedback before the pretest was conducted.

The adapted intervention materials were pretested over one summer (2024) with 10 families who had young children with SCD. The pretest involved providing families with all adapted intervention materials and delivering all adapted session content as outlined in the intervention manual. After each intervention session, qualitative feedback was gathered from participants (e.g., “What do you understand from these materials?” and “What do you especially like/dislike?”). The responses were summarized and presented to the planning group. Consistent with the approach for the initial adaptations, adaptation ideas were generated in smaller group discussions and then later presented to the larger group for voting. The final adaptations to the intervention were made after unanimous approval.

### Task 5: Plan for implementation

Previous implementation protocols for the original intervention were reviewed, and changes to the implementation of the intervention for our setting were defined. The scope and sequence of the implementation was outlined in a protocol for a pilot trial of the intervention to assess its initial feasibility and acceptability. The future implementation of the adapted intervention in our setting and at other sites is dependent on the results of the pilot trial. Training and consultation for intervention implementers was outlined and iteratively refined.

### Task 6: Plan for evaluation

The primary components of the evaluation plan were determined during the initial needs assessment and refined in this task. This involved writing evaluation questions for the adapted intervention, choosing measures to capture these outcomes, and developing the study design. During the adaptation process, refinements were made to the evaluation plan to reflect the needs of the community partners and accommodate additional outcomes of interest that were raised by caregivers and stakeholders.

## Results

### Task 1: Needs assessment

**Figure 1:**
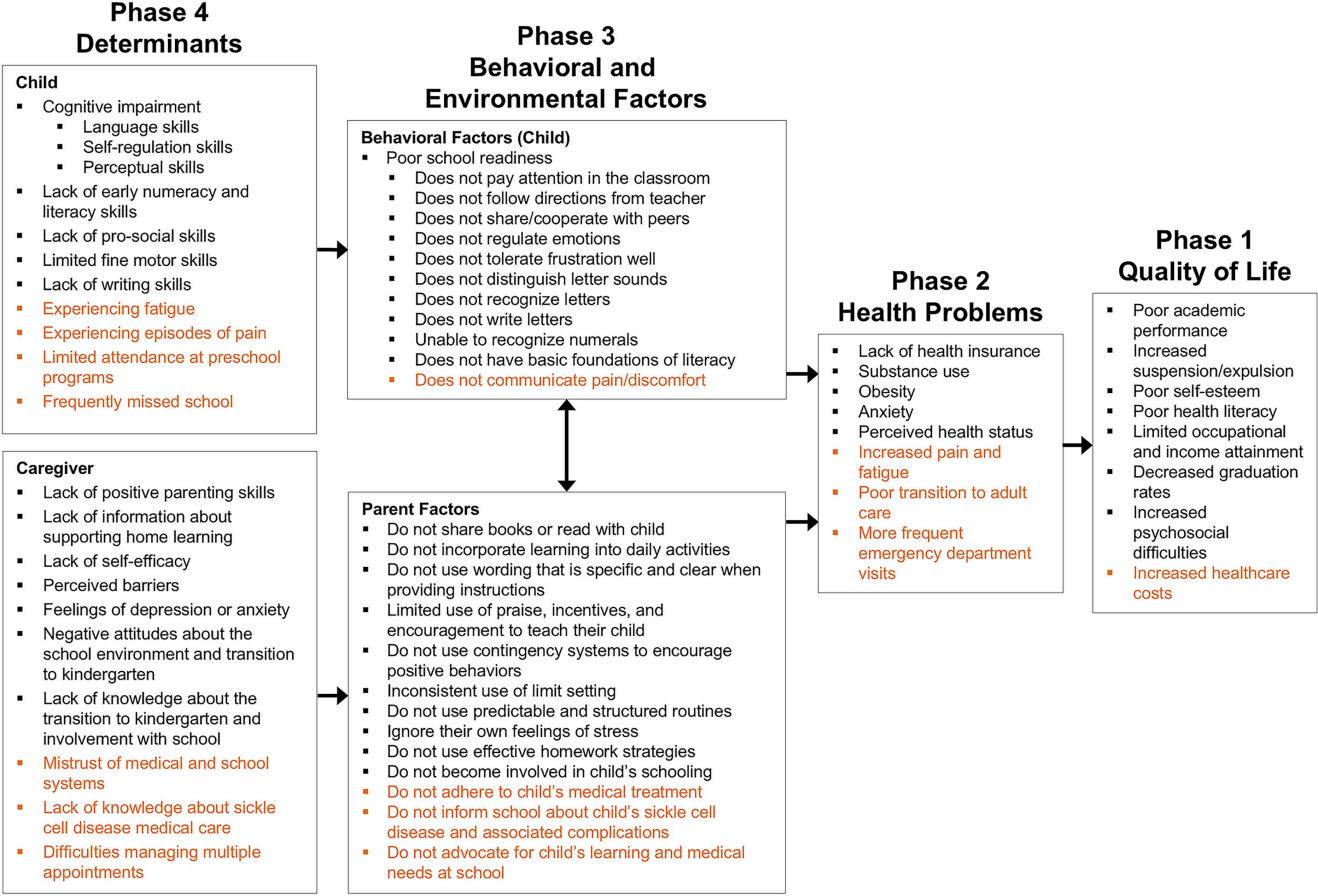
Logic model of the problem for preschool children with sickle cell disease and their parents. The original logic model for Kids in Transition to School is in black text. Adaptations specific to the sickle cell disease population are noted in orange text.

The logic model of the problem is displayed in Figure 1. Determinants and behavioral factors were divided into child and parent categories. Many determinants, behavioral/environmental factors, health problems, and quality of life outcomes for families with SCD in our setting were consistent with the original KITS intervention. Child determinants specific to SCD included the experience of fatigue, episodes of pain, limited attendance at preschool programs, and frequently missed school. SCD-related health problems resulting from these behaviors and determinants included increased pain and fatigue, poor transition to adult care, and more frequent emergency department visits.

After developing the initial model, we incorporated the findings from the mixed-methods analyses. These results have been described elsewhere (65). Briefly, both caregivers and providers indicated that positive caregiver–provider relationships had a large influence on the quality of previous intervention experiences, and such relationships enabled families to overcome mistrust of medical and school systems (determinant). Caregivers of young children with SCD showed limited knowledge of child development and several medical complications associated with SCD (determinant). Providers identified these knowledge gaps as barriers to access and medical guidance adherence (behavior). Caregivers reported difficulties managing multiple appointments for their child and other family members that often interfered with other priorities (determinant). Caregivers expressed concerns about communicating their child’s physical needs to teachers and how to ensure their safety in a larger classroom (behavior) (65).

**Figure 2:**
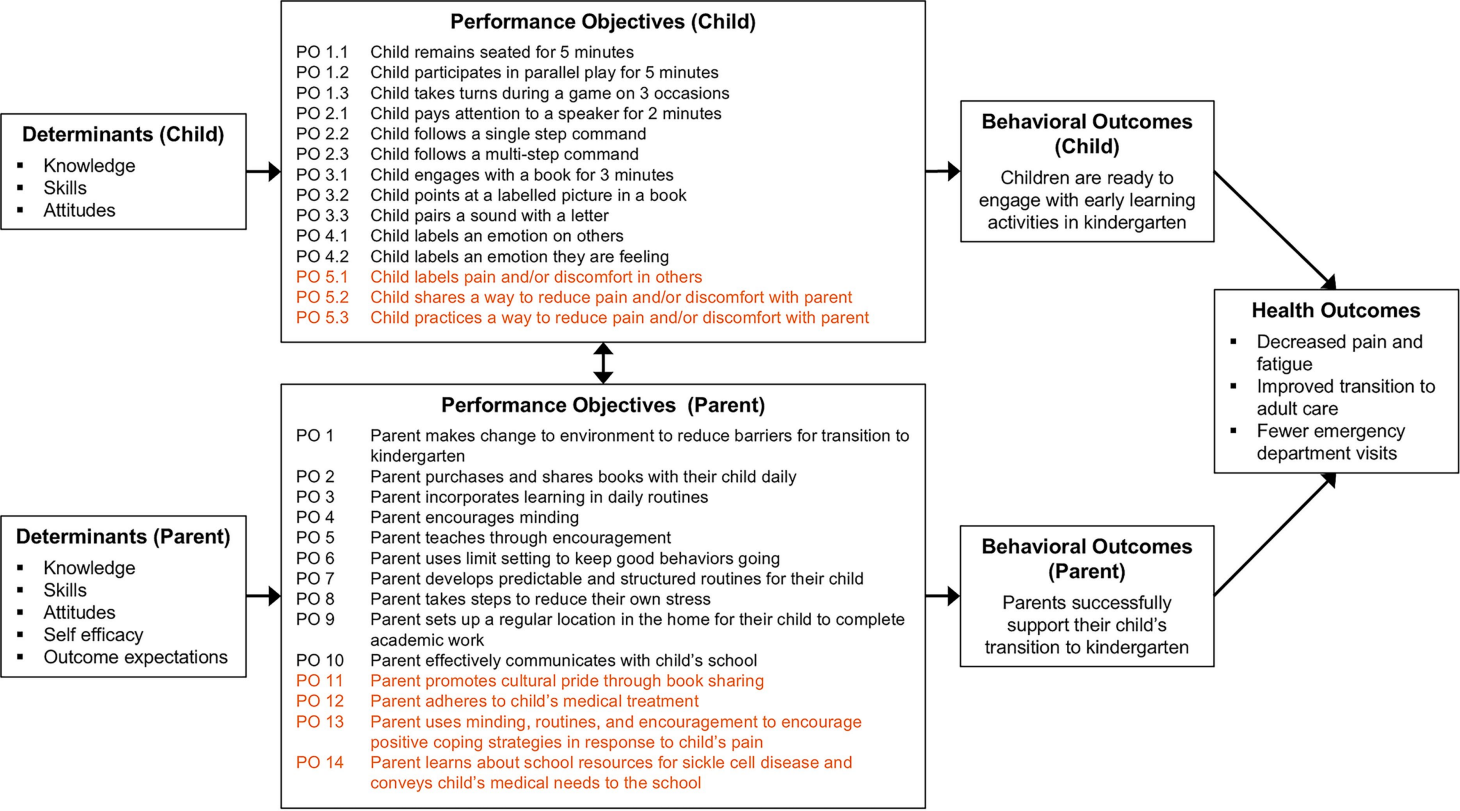
Logic model of change for the adapted school readiness intervention. Adaptations specific to the sickle cell disease population are noted in orange text.

The logic model of change is displayed in Figure 2. The determinants of behavior change for the child and parent were consistent with the original KITS intervention. For the children, these included knowledge (i.e., learning the content), skills (i.e., displaying the behavior), and attitudes (i.e., having positive feelings about the objective). For parents, determinants of behavior change included knowledge, skills, attitudes, self-efficacy (i.e., believing that they can perform the behavior), and outcome expectations (i.e., thinking that the behavior will lead to the intended outcome). Performance objectives unique to the SCD population were built from the determinants and environmental factors in the logic model of the problem (e.g., the parent learns about school resources for SCD and conveys the child’s medical needs to the school).

### Task 2: Search for evidence-based interventions

The KITS (54) intervention was chosen based on our needs assessment and the basic fit. First, the intervention showed evidence of efficacy in randomized controlled trials with predominantly low-income children raised in foster care (68) and those with developmental disabilities (41). Families of children with SCD tend to come from lower socioeconomic status backgrounds, and neurodevelopmental deficits experienced by children with developmental disabilities are also experienced by children with SCD. Second, unlike other preschool programs, a primary target of KITS is self-regulation, which is a core deficit in preschoolers with SCD (20, 21). Third, KITS has programming dedicated to teaching caregivers positive parenting strategies and provides parents with age-appropriate materials for their children. The focus on parenting skills and provision of resources is essential to address the social determinants in the home environment that are strongly associated with school readiness skills in young children with SCD (18, 19). Lastly, the curriculum for parents was implemented through both in-person and virtual formats, enabling increased flexibility and the potential to reduce barriers for families.

### Task 3: Assess fit and plan adaptations

Table 2 displays the assessment of fit between the original KITS intervention and the planned adapted intervention based on a comparison of the developed logic models. Most fit categories showed adequate or good fit. As noted in Task 1, most of the determinants, behaviors, and environmental factors were similar between the original and adapted intervention. Differences were related to SCD disease complications and population demographics.

The two areas with poor fit were the delivery method for children and the cultural relevance of the intervention. Regarding the delivery method, the original KITS intervention used in-person child groups to directly teach school readiness skills (e.g., early literacy, numeracy, self-regulation). Based on interviews with caregivers and providers, we determined that these in-person sessions were not feasible for the families to attend due to multiple barriers (e.g., transportation, lack of time and resources). Regarding cultural relevance, the individuals displayed in the KITS materials and books provided to families were not representative of the demographic makeup of our target population, which is predominantly African American.

### Task 4: Make adaptations

Adaptations to the parent group content involved incorporating components relevant to the medical complications experienced by children with SCD. This content aimed to address a lack of knowledge about SCD-related developmental differences and medical complications. Specifically, new content addressed how to respond to a child’s pain episode, the impact of sleep disruptions on school performance, medical accommodation offered to children with SCD in the school setting, and how to establish school services. We also added content regarding SCD-related neurocognitive deficits and ways to potentially limit these deficits (e.g., medical adherence). We converted the core child-directed content that was previously presented during in-person classroom sessions into animated videos for the children and their caregivers to watch at home together. Some of these videos had previously been created for a virtual version of the KITS intervention and others were specifically designed for the SCD population. In addition, we provided all families with a school readiness workbook that covered early literacy and numeracy topics. This adaptation sought to address parental concerns about managing multiple appointments and attending regular in-person visits with their child. We added child-directed content to teach children basic techniques to convey their feelings, particularly when they were fatigued or in pain. This adaptation addressed patient’s potential difficulties communicating pain/discomfort in a school setting. We updated the parent- and child-directed intervention materials to be more culturally representative by including pictures/animations of individuals from a wider range of races and ethnicities. We added books that promoted cultural pride (i.e., books featuring positive portrayals and successes) for minority populations. The adapted intervention was titled Supporting Kids in Transition to School with Sickle Cell (SKITS).

Lastly, we recruited two caregivers of young children with SCD to co-facilitate the parent group sessions in collaboration with the two interventionists recruited from the hospital’s school program. We included these caregivers to directly address feedback from families who stated that they wanted to learn the content from other caregivers with similar experiences. Families noted that they would value the input of other caregivers based on their own lived experiences more than that of providers with specialized training in child development and/or SCD. Although some of the added content was delivered directly by these facilitators, discussion questions were also used to encourage peer-to-peer problem solving.

These initial adaptations were then pretested among 10 families with children who have SCD. Caregivers ranged from 22 to 41 years (Median = 30 years), and children ranged from 4 to 5 years (Median = 4 years). All caregivers and children identified as Black/African American. Caregivers expressed strong satisfaction and perceived benefit from the intervention. Caregivers noted that group discussion and interactions were the most engaging aspect of SKITS and encouraged spending more time in discussion versus content delivery and instruction. Select sections of content were difficult for caregivers to follow (encouraging minding and pre teaching). Some caregivers reported difficulties consistently accessing the videos due to technological barriers (e.g., managing passwords, network restrictions). Facilitators reported unclear expectations around which content should be delivered by the interventionists, and which should be delivered by parent-facilitators. Feedback from the pretest was discussed at recurring meetings amongst the planning group. Several modifications were considered, and only the modifications with group consensus were incorporated. These include: (1) additional discussion questions were added to each session to facilitate more group interactions, (2) sections of the clinician manual were streamlined to reduce wordiness and increase clarity, (3) videos and handouts were added to the original KITS website to improve ease of access, and (4) the role of the parent-facilitator was more explicitly defined and delineated in the clinician manual.

### Task 5: Plan for implementation

The adapted intervention is currently being assessed in an ongoing pilot trial.^1^ The pilot trial is set to enroll 36 families, with 2:1 randomization (2 intervention: 1 control) over two summers in 2025 and 2026. The caregiver group sessions occur once weekly for 8 weeks. A virtual video platform is used to facilitate all group sessions. All families are provided physical copies of the intervention handouts and materials. Video content and copies of all handouts are hosted on the original KITS website. Families are provided with a login to view all content, and this profile keeps track of their progress (e.g., number of videos watched) throughout the intervention. Wifi hotspots are provided to all families that request internet support.

The interventionists received formal training and fidelity monitoring from the KITS author (KP) and her research team during the intervention pretest. Consistent with the original program, the interventionists are professionals trained in childhood education. The two caregiver co-facilitators were trained in the intervention alongside the other interventionists. The same training, coaching, and fidelity monitoring strategies used in the original KITS intervention were applied to all group leaders and facilitators. For training, all providers read the full clinician manual and then participated in an 6-h virtual training led by a member of the original KITS team. The training involved reviewing the core components of the intervention, group management strategies, and role-playing content from the clinician manual. Subsequently, the trainer reviewed video recordings of all sessions, completed a fidelity checklist, and provided weekly feedback and supervision to group leaders. Group leaders and caregiver co-facilitators spend approximately 3 h a week preparing for and leading a session during the first year. Time commitments progressively decrease during subsequent groups as leaders become more familiar with the content. Group leaders and co-facilitators are considered fully trained and independent after delivery of all intervention materials to two groups (i.e., 16 total sessions) and passing all fidelity checks. Subsequently, a group leader at each site is trained to be on-site trainer to facilitate future implementation.

The content of each session is split between the interventionists and parent facilitators. The interventionists focus on teaching the core concepts of SKITS, and the parent facilitators discuss how to apply the material to the lived experience of raising a child with SCD.

An institutional protocol outlines the pilot trial’s scope and sequence, including the study objectives, participant eligibility, design, evaluations, risks, and data collection methods. The primary objective of the pilot trial is to assess the initial feasibility and acceptability of SKITS. Secondary and exploratory objectives include comparing the efficacy of SKITS to that of routine care and identifying factors that influence SKITS implementation.

### Task 6: Plan for evaluation

The primary outcomes of the pilot trial are the feasibility and acceptability of SKITS. Feasibility will be measured through quantitative metrics, including the ratio of consented to approached families and the percentage of sessions completed (caregiver and child). Acceptability will be assessed through semi-structured, post-intervention interviews with caregivers. These interviews will assess affective attitude, burden, ethicality, intervention coherence, opportunity costs, perceived effectiveness, and self-efficacy from the Framework of Acceptability (69). Caregivers and intervention providers will complete the Feasibility of Intervention Measure and Acceptability of Intervention Measure post-intervention (70).

Secondary outcomes include metrics of efficacy and will be assessed pre- and post-intervention. These measures assess early children’s literacy, numeracy, self-regulation, inhibitory control, and social skills, along with caregivers’ parenting skills, emotional regulation, and self-efficacy. These tools were chosen to be consistent with the logic model of change (Figure 2).

Lastly, to examine implementation factors (i.e., barriers and facilitators), we will conduct semi-structured, post-intervention interviews with interventionists and 10 providers/stakeholders at St. Jude. Consistent with the initial adaptation process, interview guides will be based on the HEIF (58).

The decision to proceed to a future trial or to make further adaptations will be based on *a priori* feasibility criteria. If all three criteria are not met, we will examine caregiver and providers/stakeholder data (collected before and after the pilot trial) to inform the next steps. Potential steps include further adaptation or applying the knowledge gained to the adaptation of an alternative intervention. If feasibility criteria are met, we plan to develop a multi-site hybrid implementation-effectiveness trial of the adapted school readiness intervention.

## Discussion

We have used the IM Adapt approach to inform the adaptation, implementation, and evaluation of a school readiness intervention for preschool-aged children with SCD. Through systematic adaptation, we learned about the unique needs and preferences of patients with SCD and their families and adapted KITS accordingly. Community engagement was emphasized at all stages of the adaptation.

Multiple behaviors and environmental conditions of an intervention can be modified using IM Adapt. For example, this methodology has been used to adapt an intervention to stimulate physical activity in disabled people (71) and a weight management program for Latinx children (72). Consistent with this previous work, SKITS targets many parent and child behaviors that are associated with school readiness and potential health problems later in life. SKITS aims to improve the school readiness of children with SCD. Within the IM Adapt framework, school readiness is conceptualized as a behavioral and/or environmental factor that contributes to subsequent health problems and quality of life. Therefore, when we developed the original logic models, we conceptualized school readiness components as performance objectives (Figures 1, 2). This process informed which parent and child behaviors from the original KITS were a poor fit for SKITS. These experiences and the associated models may assist other investigators who seek to use IM Adapt for other neurobehavioral or educational interventions.

A major adaptation involved shifting the content delivery from child classroom sessions to animated videos. The goal of these videos is to facilitate parent–child interactions and practice of the skills/concepts learned in the parent sessions. Learning from children’s peer interactions or following directions in a classroom setting cannot be captured in a virtual format. However, we provide families with local resources to facilitate peer interactions of the child in a classroom setting. Some of these videos were specifically designed for the added content from SKITS and others were developed due to a need to shift the original intervention to a virtual format during the COVID-19 pandemic. The KITS team has conducted the intervention virtually for several years and now offers the option of either an in-person or virtual approach. Although the transition to virtual instruction increases accessibility, it is unknown if the virtual delivery attenuates the effect that the intervention has on children’s school readiness skills through direct instruction in a classroom setting. Further research is needed to evaluate the sensitivity of outcome measures (e.g., performance-based measures vs. parent report) based on the virtual delivery.

Including parents of children with SCD as intervention co-facilitators addressed multiple fit categories. First, these parents could relate to the lived experience of SCD that many parents felt was lacking in their previous encounters with medical professionals. Second, the co-facilitator could promote community-building among families of children with SCD by enabling them to interact in a group setting. Lastly, the co-facilitators share many cultural and demographic features with the target population, which enables them to relate to the sociodemographic factors that influenced their experience with SCD. Similar adaptations, such as matching a client and behavioral therapist based on race, have shown variable efficacy, with the greatest effect sizes observed for African American patients (73). Broadly, these strategies are used to address patient preference and increase patient-provider rapport (74). Incorporating individuals with shared medical histories as behavioral intervention providers has been used in previous treatments for patients with SCD^76^ and HIV (75, 76). These strategies have shown potential but require further research to determine whether such adaptations improve intervention engagement or efficacy (77). Due to the group format and virtual delivery, the overall costs and training demands are thought to be relatively low and highly feasible for most centers serving patients with SCD, although no formal cost-analysis has yet been performed. Patient-facing materials (e.g., handouts and videos) are easily accessible to families from the KITS website. If pilot trial findings are positive, future work will examine if SKITS is sustainable over time or at a larger scale of implementation.

Using the HEIF framework, we will explore factors that affect the implementation and health equity of SKITS within our setting during the pilot trial (58). These include the group session encounters, patient factors, provider factors, and characteristics of the adaptation. We will also examine the influence of broader contextual factors, including the hospital system, local community, and sociopolitical influences. These findings will influence subsequent iterative adaptations of the intervention and inform the planning for its future implementation at a larger scale. Despite substantial adaptations to improve the intervention implementation, many barriers remain, as documented by previous efforts to implement behavioral interventions for this population (35, 78). It is unlikely that one school readiness intervention will be a good fit for all families with SCD, so multiple approaches may be required.

There are several limitations to the adaptation of SKITS. First, the participants interviewed and surveyed for the needs assessment lived in one Southern city in the US. Patients were served by a well-resourced academic medical center with an integrated school program. The needs and associated adaptations for this group may be limited to this geographic region and similarly resourced hospitals. Second, there is no published data on the virtual implementation of the original intervention (KITS) to enable direct comparison with SKITS. However, these data have been collected and are forthcoming. Strengths of the adaptation include the use of a theory-driven approach that clearly delineates the rationale for adaptations, the steps taken, and the intervention components that were modified. This process allows for future iterative changes and the application of findings to other similar interventions. In addition, consistent community engagement throughout the adaptation fostered new relationships among SCD community members and enabled them to contribute to additional programming for the SCD population at our center.

## Conclusion

We used IM Adapt to adapt an evidence-based school readiness intervention for children with SCD. By working with families and community stakeholders, we may be able to adapt evidence-based interventions to address barriers and health inequities, thereby strengthening the implementation of services.

## Data Availability

The datasets presented in this article are not readily available because data sharing is not applicable to this article as no datasets were generated or analyzed during the current study. Requests to access the datasets should be directed to andrew.heitzer@stjude.org.

## Footnotes

1
https://clinicaltrials.gov/study/NCT06367192
